# Insights from transcriptome profiling on the non-photosynthetic and stomatal signaling response of maize carbonic anhydrase mutants to low CO_2_

**DOI:** 10.1186/s12864-019-5522-7

**Published:** 2019-02-15

**Authors:** Allison R. Kolbe, Anthony J. Studer, Omar E. Cornejo, Asaph B. Cousins

**Affiliations:** 1 School of Biological Sciences, Washington State University, Pullman, WA USA; 2 Department of Crop Sciences, University of Illinois Urbana-Champaign, Urbana, IL USA

**Keywords:** Carbonic anhydrase, C_4_ photosynthesis, Low CO_2_, RNA-seq, Stomata, *Zea mays*

## Abstract

**Background:**

Carbonic anhydrase (CA) catalyzes the hydration of CO_2_ in the first biochemical step of C_4_ photosynthesis, and has been considered a potentially rate-limiting step when CO_2_ availability within a leaf is low. Previous work in *Zea mays* (maize) with a double knockout of the two highest-expressed β-CA genes, CA1 and CA2, reduced total leaf CA activity to less than 3% of wild-type. Surprisingly, this did not limit photosynthesis in maize at ambient or higher CO_2_concentrations. However, the *ca1ca2* mutants exhibited reduced rates of photosynthesis at sub-ambient CO_2_, and accumulated less biomass when grown under sub-ambient CO_2_ (9.2 Pa). To further clarify the importance of CA for C_4_ photosynthesis, we assessed gene expression changes in wild-type, *ca1* and *ca1ca2* mutants in response to changes in *p*CO_2_ from 920 to 9.2 Pa.

**Results:**

Leaf samples from each genotype were collected for RNA-seq analysis at high CO_2_ and at two time points after the low CO_2_ transition, in order to identify early and longer-term responses to CO_2_ deprivation. Despite the existence of multiple isoforms of CA, no other CA genes were upregulated in CA mutants. Although photosynthetic genes were downregulated in response to low CO_2_, differential expression was not observed between genotypes. However, multiple indicators of carbon starvation were present in the mutants, including amino acid synthesis, carbohydrate metabolism, and sugar signaling. In particular, multiple genes previously implicated in low carbon stress such as asparagine synthetase, amino acid transporters, trehalose-6-phosphate synthase, as well as many transcription factors, were strongly upregulated. Furthermore, genes in the CO_2_ stomatal signaling pathway were differentially expressed in the CA mutants under low CO_2_.

**Conclusions:**

Using a transcriptomic approach, we showed that carbonic anhydrase mutants do not compensate for the lack of CA activity by upregulating other CA or photosynthetic genes, but rather experienced extreme carbon stress when grown under low CO_2_. Our results also support a role for CA in the CO_2_ stomatal signaling pathway. This study provides insight into the importance of CA for C_4_ photosynthesis and its role in stomatal signaling.

**Electronic supplementary material:**

The online version of this article (10.1186/s12864-019-5522-7) contains supplementary material, which is available to authorized users.

## Background

Carbonic anhydrase (CA) is a ubiquitous enzyme across all kingdoms of life, catalyzing the reversible hydration of CO_2_ into bicarbonate [1]. In plants, the β-subtype of CA is often present in large quantities and has long been implicated in photosynthesis [2]. The role of β-CA in C_3_ plants remains somewhat ambiguous, with reports that CA makes up a significant portion of leaf protein, but β-CA knockdowns show little photosynthetic phenotype [3, 4]. In C_3_ plants, β-CA is primarily found in the chloroplast and the prevailing hypothesis is that it facilitates diffusion of CO_2_ across the chloroplast envelope [1]. In contrast, β-CA is primarily localized to the mesophyll cytosol in C_4_ plants, where it produces bicarbonate for phosphoenolpyruvate carboxylase (PEPC) in the first committed step of the CO_2_ concentrating mechanism.

Various modeling studies and mutant analyses have been employed to assess the role of β-CA for C_4_ photosynthesis. For example, modeling the apparent in vivo CA activity extrapolated from in vitro enzyme assays suggested that CA may be near-limiting for C_4_ photosynthesis [5]. Subsequent mutant analyses in the C_4_ dicot *Flaveria bidentis* found that CA was in excess for photosynthesis, as CO_2_ assimilation was unaffected until CA levels reached < 20% of wild-type [6, 7]. However, C_4_ monocots such as maize had previously been shown to have naturally low levels of CA activity [8], suggesting that CA may be limiting in these plants. To address this question, Studer et al. [9] knocked out the two highest-expressed β-CA genes in maize (*Ca1* and *Ca2*). Surprisingly, a 97% reduction of wild-type CA activity resulted in plants capable of maintaining wild-type rates of photosynthesis at ambient and above partial pressures of CO_2_ (*p*CO_2_), with significant reductions in photosynthesis occurring only under sub-ambient *p*CO_2_. RNAi knockdowns of CA to ~ 10% of wild-type in *Setaria viridis* showed similar results [10]. Taken together, it appears that CA activity is in excess for what is needed to drive photosynthesis in C_4_ plants under ambient *p*CO_2_. However, these results do not provide a satisfactory explanation for the overabundance of CA in C_4_ leaves, and also do not indicate if CA mutants are able to compensate through alternate means of enhancing CO_2_ diffusion and bicarbonate availability.

In addition to photosynthesis, CA has been shown to serve diverse roles in plant metabolism. Isoforms of α and γ CA subtypes identified in plants are primarily expressed in non-photosynthetic tissue. The function of α-CA in higher plants is not known [11]. Similarly, the role of γ-CA is not well understood, although mutant analyses in *Arabidopsis thaliana* have implicated γ-CAs in the assembly of Complex I in the mitochondrial electron transport chain and suggested roles for γ-CAs in light-dependent development and photorespiration [12–14]. Although β-CAs are primarily considered for their photosynthetic role, isoforms of β-CAs in C_3_ plants have also been implicated in lipid biosynthesis [15], stomatal responses to CO_2_ [16, 17], recapture of photorespired CO_2_ [14, 18], and amino acid biosynthesis [19]. Furthermore, recent work in C_4_ plants has suggested a link between β-CA, nitrogen metabolites, and photosynthesis [20], and a role for β-CA in stomatal movement [21]. Therefore, β-CAs may serve in a variety of roles in C_4_ plants.

To further evaluate the role of β-CA in the C_4_ plant maize, RNA-seq was performed on *ca1* and *ca1ca2* mutants to test the transcript-level effect of CA limitation under high and low *p*CO_2_. We hypothesized that CA mutants would upregulate other CA isoforms, photosynthetic genes, or genes influencing CO_2_ diffusion into the leaf in order to compensate for the CA limitation. This experiment also provided an opportunity to evaluate the potential role of β-CA in non-photosynthetic roles in a C_4_ plant. Overall, our results provide new insights into the role of β-CA in maize and mechanisms of low CO_2_ adaptation in CA-limited plants.

## Results

RNA-seq was performed on wild-type and *ca1* and *ca1ca2* mutant alleles (described previously in [9]) in order to evaluate the effect of CA limitation on transcriptional responses to low CO_2_. Plants were initially grown at 920 Pa (10,000 ppm) CO_2_ as previously described [9] to minimize growth differences between genotypes. To verify that genotypes were similar under the high CO_2_ growth conditions, leaf samples were taken for RNA-seq after 12 days at 920 Pa. After the CO_2_ levels were reduced to 9.2 Pa (100 ppm), two additional time points were sampled to capture rapid and longer-term response differences between genotypes to reduced CO_2_ availability.

**Fig. 1:**
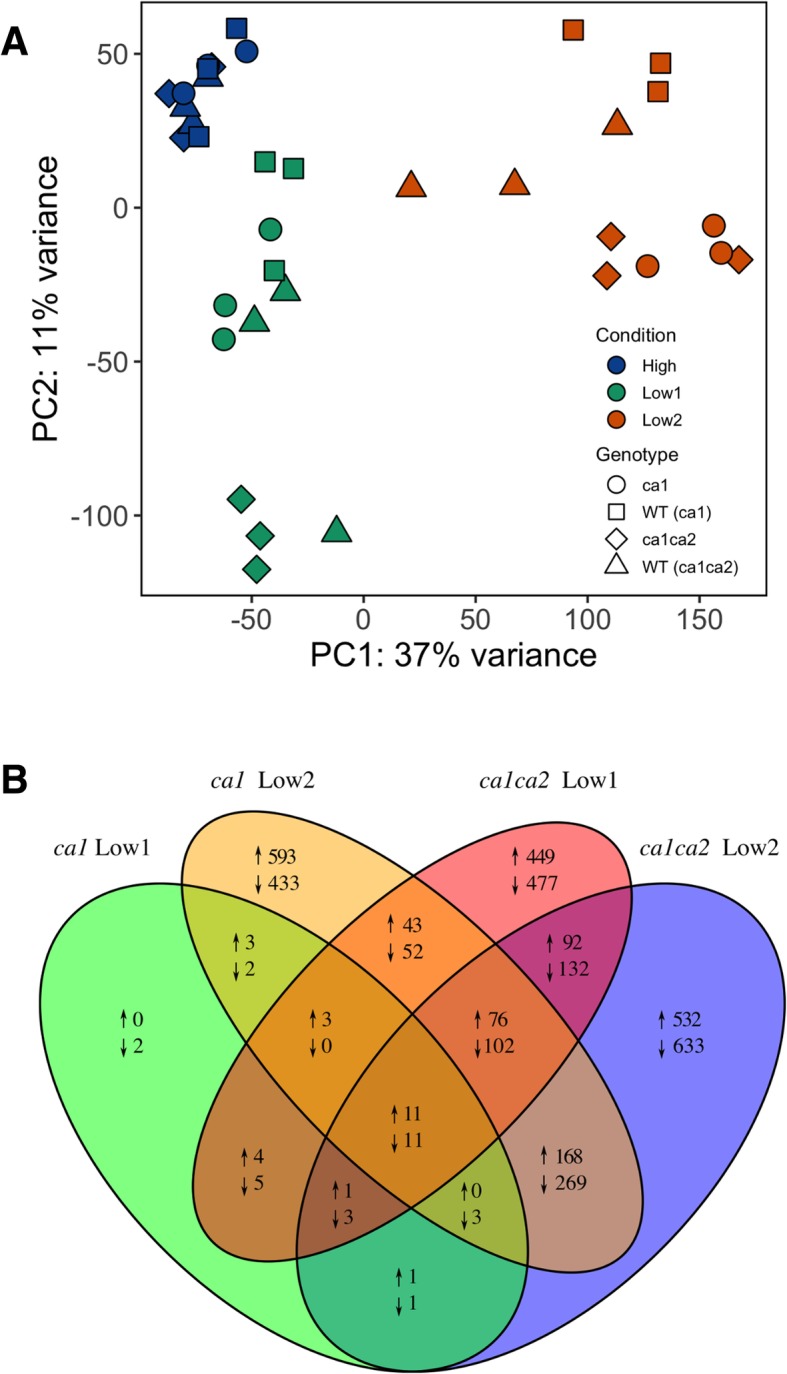
Principal component analysis of log-transformed reads (**a**) and Venn Diagram indicating statistically significant differentially expressed genes (**b**) in both low CO_2_ time points in *ca1* and *ca1ca2* mutant plants relative to wild-type. Differential expression was determined by an FDR cutoff of 0.05 using the quasi-likelihood negative binomial generalized log-linear model in edgeR

Principal component analysis of log_2_-transformed reads showed that the CO_2_ treatment had the strongest effect on gene expression (PC1: 37%), whereas the genotype corresponded to the second principal component (11% of variance) (Fig. 1a). All genotypes were tightly clustered at high CO_2_ (“High”), consistent with previous findings that high CO_2_ rescues the CA mutant phenotype [9]. Under high CO_2_ conditions, *ca1ca2* mutant plants had 170 significantly differentially expressed genes, whereas the *ca1* mutant plants had 42 differentially expressed genes. Of these, 19 differentially expressed genes were shared between the genotypes. Gene ontology (GO) term enrichment found no significant GO term enrichment in *ca1* mutants at high CO_2_, but the *ca1ca2* mutants were enriched in genes involved in regulation of gene expression (GO:0010468).

The *ca1ca2* mutants were more strongly affected by the first day at low CO_2_ than *ca1* mutant plants, as evidenced by their separation in the PCA as well as the number of differentially expressed genes (Fig. 1b). Again, the differentially expressed genes of *ca1* mutants were not significantly enriched in any GO terms; however, *ca1ca2* mutants were enriched in many stress responses, signaling, carbohydrate metabolism, and cellulose biosynthesis genes. After two days at low CO_2_ (“Low2”), both *ca1* and *ca1ca2* mutants were clearly distinct from wild-type in the PCA and many genes were differentially expressed (Fig. 1b). In both mutant genotypes, the differentially expressed genes at Low2 were enriched in GO categories including carbohydrate metabolism, cellulose biosynthesis, trehalose biosynthesis, stress responses, epidermis development, and organic acid metabolism (Additional file 1).

Five genes were differentially expressed in the *ca1* and *ca1ca2* mutants across all conditions. However, these five genes were unresponsive to CO_2_, and located on the same chromosome as *ca1* and *ca2* (chromosome 3). Differential expression of these genes was determined to be an artifact of backcrossing the W22-based mutant into a B73 background. Due to linkage, homozygous mutant plants have W22 alleles of genes adjacent to the *Ca* locus and the wild-type plants have B73 alleles. The differential expression of these five genes in a W22 and B73 background is consistent with the expression measured by [22].

Transcripts accumulated for both *ca1* and *ca2* in the mutant genotypes as expected due to the location of the *Ds* element in *ca2* and the footprint allele in *ca1* [9], although in most cases *ca1* and *ca2* expression was statistically reduced in the mutants (Table 1). However, previous work showed significant reductions in CA activity in *ca1* and *ca1ca2* mutants [9] despite transcription of the majority of the gene. As previously described in Studer et al. [9], *Ca3* was incorrectly annotated as part of *Ca2* in the AGPv3 assembly. However, *Ca3* is lowly expressed in leaf tissue, and visualization of the gene region in IGV shows no up-regulation of the gene in *ca1* or *ca1ca2* mutants (Additional file 2). The apparent down-regulation of *Ca3* in mutant plants is most likely artefactual and due to misalignment of *Ca1* and *Ca2* sequences, as *Ca3* has very high sequence homology to these other CA genes. Since fewer *Ca1* and *Ca2* transcripts are present in mutant plants, there are fewer reads aligning to *Ca3* as well. Although there are three additional genes encoding β-CAs in maize leaf tissue, none of these were up-regulated in the mutants (Table 1). Consistent with previous reports showing no change in activity of the key photosynthetic enzymes PEPC or Rubisco in *ca1* or *ca1ca2* mutant plants compared to wild-type [9], there was no evidence of compensation in the expression of C_3_ or C_4_ cycle components in either mutant genotype, regardless of the *p*CO_2_ during the sampling. In fact, when significant differences were observed, these genes tended to have lower expression compared to wild-type at low CO_2_ (Additional file 3). Similarly, genes encoding photorespiratory enzymes were not differentially expressed between mutants and wild-type plants, although these genes tended to be downregulated at low pCO_2_ in all genotypes_._

**Table 1:** Log-fold change and significance values of β-carbonic anhydrases found in leaf tissue in *ca1* and *ca1ca2* mutant plants at each CO_2_ condition. Significantly differentially expressed genes were defined as FDR < 0.05

		*ca1* log-fold change (FDR)			*ca1ca2* log-fold change (FDR)		
Gene ID	Predicted subcellular localization	High	Low1	Low2	High	Low1	Low2
GRMZM2G121878 (*Ca1*)	Chloroplastic/cytosolic	−0.86 (0.98)	−1.1 (0.18)	−1.9 (0.0007)	− 1.66 (0.0098)	−1.25 (0.019)	− 1.32 (0.010)
GRMZM2G348512 (*Ca2*)	Cytosolic	−0.84 (0.14)	−0.75 (0.20)	−1.0 (0.0025)	−1.97 (0.0003)	− 1.95 (0.0002)	−1.28 (0.0021)
GRMZM2G094165	Chloroplastic	0.49 (1)	0.76 (0.19)	−0.13 (0.82)	0.20 (1)	0.37 (0.50)	0.79 (0.084)
GRMZM2G414528	Mitochondrial	−0.06 (1)	0.05 (1)	0.15 (0.80)	−0.27 (1)	0.072 (0.91)	0.34 (0.43)
GRMZM2G145101	Mitochondrial	−.12 (1)	−0.06 (1)	0.18 (0.75)	−0.042 (1)	0.22 (0.65)	−0.25 (0.59)

Aquaporins have been implicated in mediating CO_2_ diffusion across membranes in various plant species [23]. However, no aquaporin genes were consistently upregulated in mutant plants at low *p*CO_2_. In *ca1ca2* mutants, *Zm*PIP1;2 (AC209208.3_FG002) was upregulated and *Zm*PIP2;5 (GRMZM2G178693) was downregulated. In contrast, only *Zm*PIP1;6 (GRMZM2G136032) was significantly differentially expressed in *ca1* mutants and was downregulated at low *p*CO_2_. Interestingly, the expression patterns of the various maize aquaporin genes in response to CO_2_ varied, with some increasing at low CO_2_ and others decreasing (Additional file 4). Expression of *Zm*PIP1;2 increased steadily in response to CO_2_, with expression in the *ca1ca2* mutants surpassing wild-type at the second low CO_2_ time point. In contrast, *Zm*PIP2;5 exhibited an expression pattern similar to the photosynthetic genes – relatively constant between the high CO_2_ and first low CO_2_ time point, but sharply reduced after two days at low CO_2_. Localization data for the mesophyll and bundle sheath provides additional insight into the potential function of these aquaporin genes. *Zm*PIP1;2 and *Zm*PIP1;6 are found in both cell types, whereas *Zm*PIP2;5 is primarily localized to the bundle sheath [24].

**Fig. 2:**
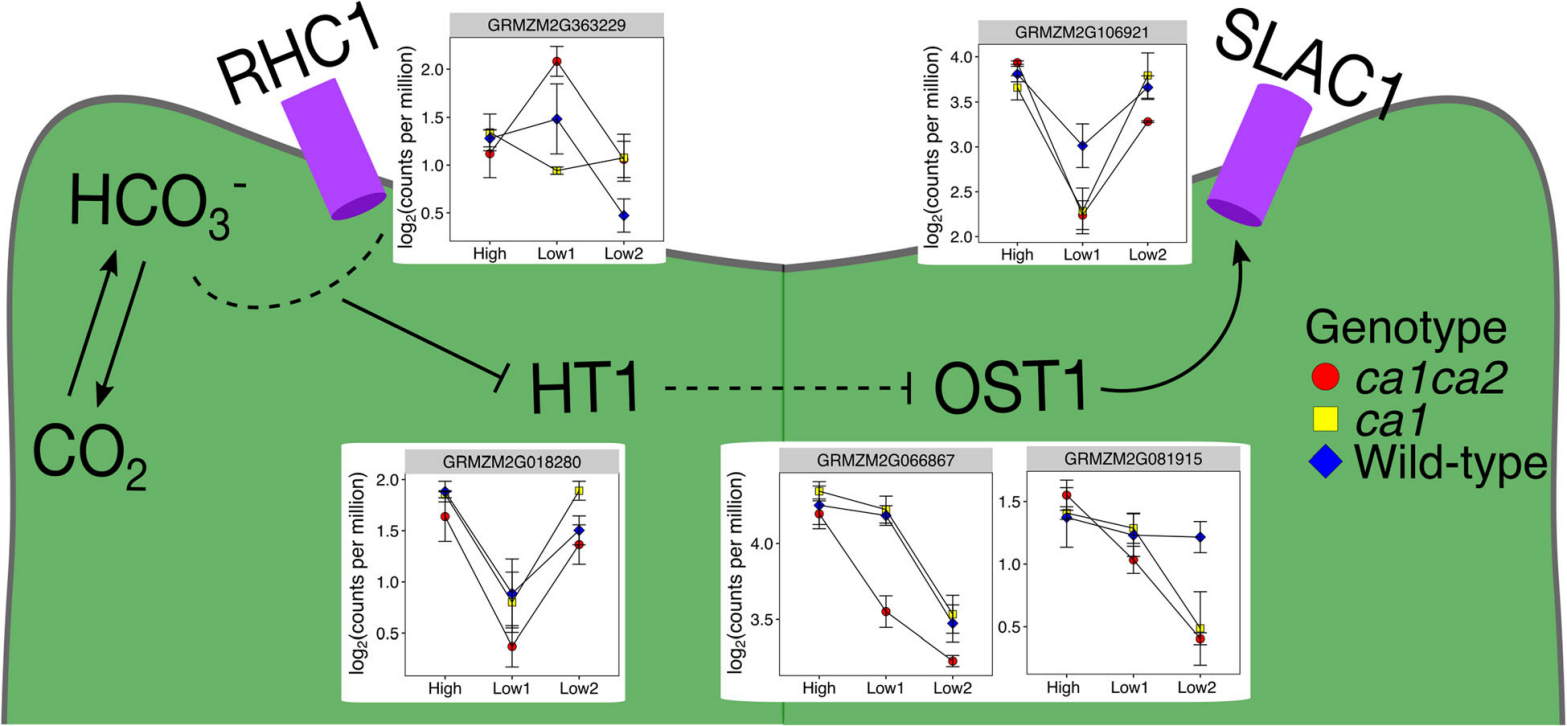
Expression patterns for genes involved in the CO_2_ signaling pathway of the stomata. Wild-type data are presented as an average of both wild-type genotypes. Data are presented as the average of log-transformed counts-per-million +/− standard error. Green area represents a maize guard cell. The bicarbonate pool produced by CA-medicated CO_2_ hydration is sensed by RHC1. When bicarbonate levels are high, RHC1 interacts with and binds HT1. This prevents HT1 from inhibiting OST1 (indicated with dashed line). With HT1 sequestered to the plasma membrane, OST1 is able to activate SLAC1, which results in stomatal closure. As pictured here, RHC1 and SLAC1 are both expected to localize to the plasma membrane [28]

Carbonic anhydrase has previously been shown to function in the stomata to initiate stomatal responses to CO_2_. The CO_2_-sensing pathway of stomata has been characterized in *Arabidopsis thaliana*, but it is not well known whether monocots or C_4_ species use a similar signaling pathway. Similar to what was previously shown in *Arabidopsis* [16], *ca1* and *ca1ca2* mutants have altered stomatal conductance and responsiveness [21]. Therefore, we hypothesized that downstream components of the CO_2_ signaling pathway are likely conserved in maize. Homologs of the genes in the CO_2_ pathway from *Arabidopsis* were identified and used to evaluate the downstream effects of CA knockouts on stomatal signaling genes. Genes downstream of CA in the stomatal CO_2_ signaling pathway altered their expression in response to changing CO_2_ as expected. In addition, several genes involved in the CO_2_ sensing mechanism of the stomata were differentially expressed between *ca* mutants and wild-type plants (Fig. 2). Other components of the CO_2_ signaling pathway, including RHC1 and HT1, responded as expected to drive stomatal opening (Fig. 2), but differential expression was not observed between genotypes.

Among the genes differentially expressed in both genotypes at low CO_2_ were several groups characteristic of carbon starvation responses. Four genes likely encoding trehalose phosphate synthase (TPS) were expressed significantly higher in both *ca1* and *ca1ca2* mutants at low CO_2_. Interestingly, these TPS genes were expressed at similar levels at high CO_2_ in all genotypes, and were generally downregulated in response to low CO_2_ in wild-type plants. However, the mutant plants either maintained or increased expression levels of several TPS genes in response to low CO_2_ (Additional file 5).

Multiple genes relating to starch and sucrose synthesis were also differentially expressed in *ca* mutants under low *p*CO_2_. In general, genes involved in the breakdown and mobilization of sucrose, including SWEET transporters and sucrose synthase, were upregulated. The sucrose synthase breakdown pathway, driven by *shrunken1* in maize, has lower energetic costs and tends to be favored under energy limited conditions [25]. Two genes encoding glucose-6-phosphate translocators were significantly downregulated in the mutants. Furthermore, genes involved in starch synthesis were likewise downregulated. Overall these results are consistent with decreased carbon supply and decreased investment into the starch and sucrose pathways in the mutant plants relative to wild-type under low CO_2_ (Additional file 4).

**Fig. 3:**
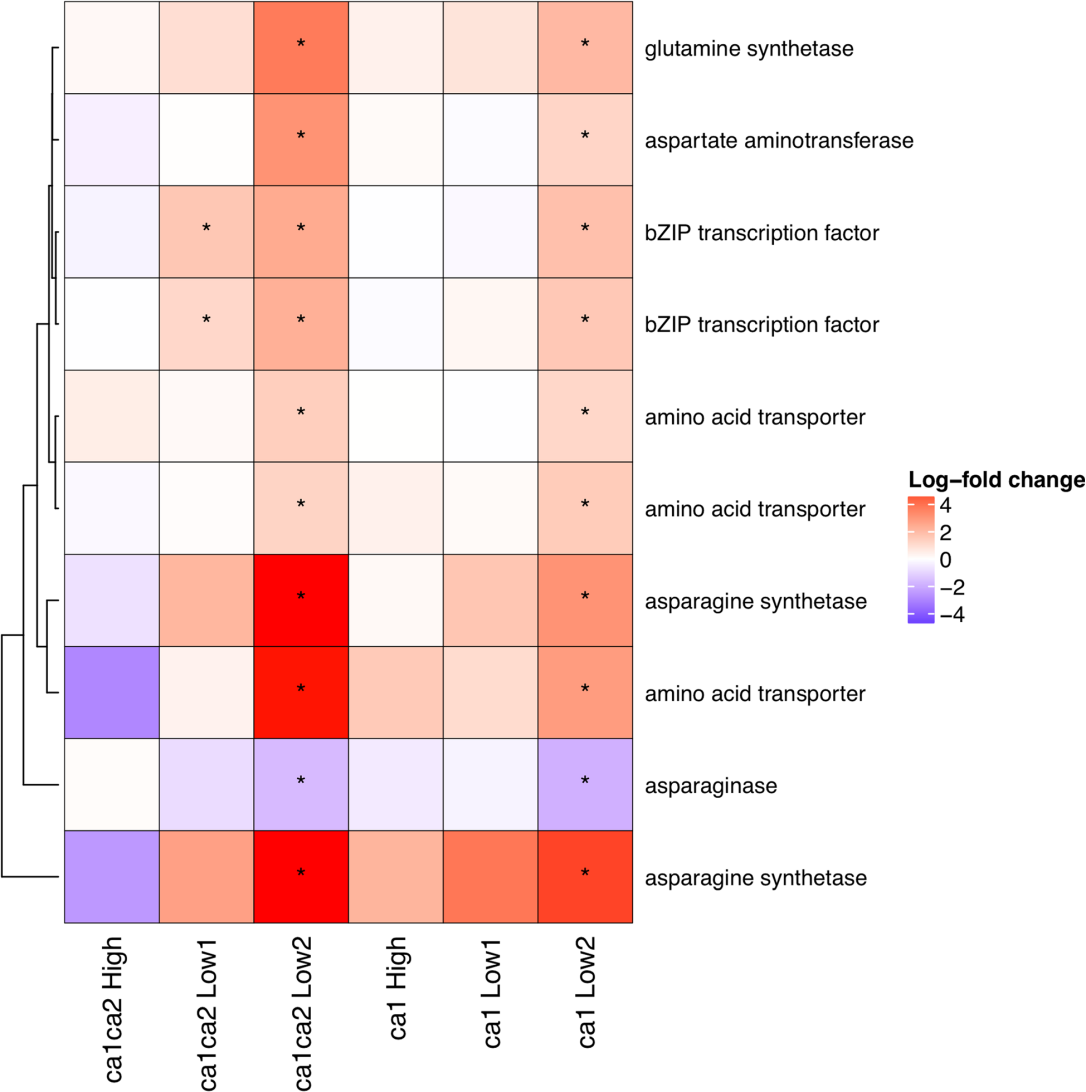
Top differentially expressed genes involved in synthesis of high N:C amino acids and remobilization. Colors indicate log-fold change relative to wild-type. Significant differences are marked with an asterisk (*). Gene IDs are given in Additional file 6

In addition to reprogramming of carbohydrate metabolism, carbon starvation is often characterized by specific changes in nitrogen metabolism. In this experiment, asparagine synthetase, aspartate aminotransferase, and glutamine synthetase were strongly upregulated under low CO_2_ in the mutants. Furthermore, several putative amino acid transporters were also upregulated at low CO_2_ (Fig. 3; Additional file 6). Interestingly, orthologs of several transcription factors associated with low energy signaling in *Arabidopsis*, including regulation of asparagine synthetase, were also upregulated in the mutants at low CO_2_. This indicates a likely parallel role for these genes in maize (Fig 3; Additional file 6).

Another metabolic process affected by low CO_2_ in the mutants, consistent with a carbon starvation response, was cell wall synthesis and growth. Four isoforms of cellulose synthase, three isoforms of β-galactosidase, and three isoforms of endoglucanase were significantly downregulated in *ca1* and *ca1ca2* mutants compared to wild-type plants at low CO_2_ (Additional file 4). Interestingly, several genes encoding homologs of expansin-like genes from *Arabidopsis* were upregulated at low CO_2_ in *ca1ca2* mutants.

**Fig. 4:**
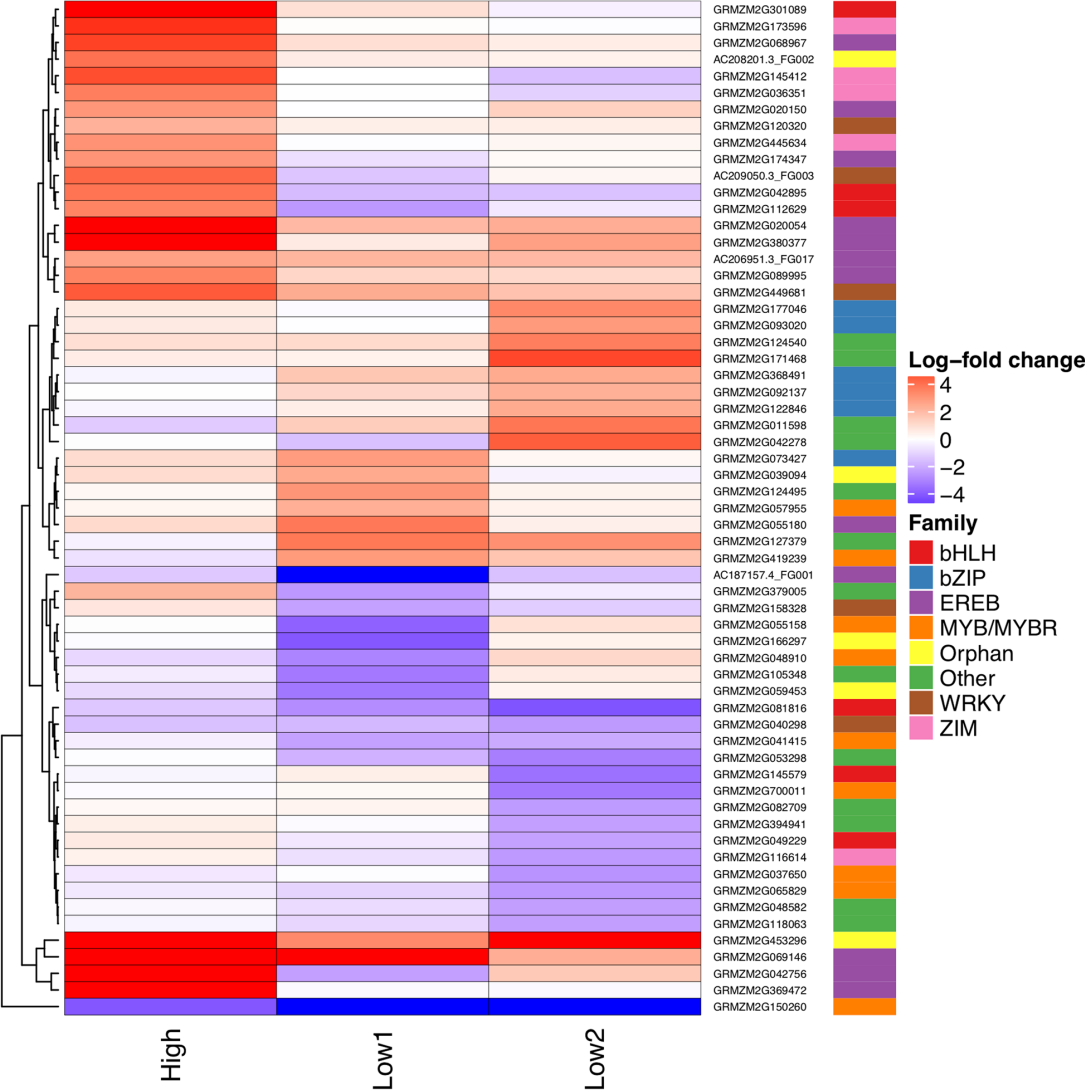
Transcription factors that were differentially expressed in *ca1ca2* mutants at FDR < 0.01, log-fold change > 2 or  < -2 , in at least one CO_2_ condition. Genes were clustered according to expression pattern. Colored bars to the right indicate transcription family membership. Families represented by fewer than 5 transcription factors were grouped into “Other”. The families for these genes can be found in Additional file 7

Differentially expressed transcription factors were identified using publicly available data from GRASSIUS [26]. A total of 324 transcription factors were differentially expressed in at least one genotype/condition. At high CO_2_, only two transcription factors were identified as differentially expressed in *ca1* mutants; these genes were likewise differentially expressed in *ca1ca2* mutants. Similarly, three transcription factors were differentially expressed in *ca1* mutants at the first low CO_2_ time point, which were all shared with *ca1ca2* mutants. However, *ca1ca2* mutants also differentially expressed many more transcription factors under high CO_2_ and the first low CO_2_ time point that were not shared with *ca1* mutants: 33 and 115, respectively. After two days at low CO_2_, *ca1* mutants had 111 differentially expressed transcription factors, *ca1ca2* mutants had 165, and 48 of these were shared. Many transcription factor families were represented in these differentially expressed genes (Fig. 4). Of the shared genes with *Arabidopsis* homologs of known function, several were involved in low energy signaling, regulation of asparagine synthetase, and stomatal regulation (Additional files 6 and 7).

Twelve genes that were found to be either differentially expressed or responsive to CO_2_ in the RNA-seq analysis were validated with qRT-PCR. The log_2_-fold change between mutant and wild-type was calculated for each condition and compared to the log_2_-fold change calculated using edgeR in the RNA-seq analysis. Similar results were observed using both methods (Additional file 8). The log_2_-fold change values generated by qPCR and RNA-seq were significantly correlated (*p* < 2e^− 16^, R^2^ = 0.82; Additional file 9). All genes which were differentially expressed in one or more condition showed similar patterns in the qRT-PCR data. Additionally, we included one gene that was not differentially expressed in the RNA-seq data (PEPC); this result was also confirmed in the qRT-PCR results. Furthermore, patterns of CO_2_responsiveness were consistent between the qRT-PCR and RNA-seq data (Additional file 10). Thus, the observed gene expression trends are supported by two orthogonal methods providing strong evidence for the observed results.

## Discussion

### CA mutants at high CO_2_

Consistent with previous observations that *ca* mutants grow normally under high CO_2_, both *ca1* and *ca1ca2* mutants clustered with wild-type plants in the principal component analysis and there were only a few genes differentially expressed between genotypes at high CO_2_. As noted by the GO enrichment analysis, the *ca1ca2* mutants differentially express many transcription factors at high CO_2_; however, these genes are uncharacterized in maize and have unknown functions. Only 19 genes were differentially expressed in both genotypes at high CO_2_, indicating that the mutations had a minor effect on gene expression at high CO_2_. Importantly, no photosynthetic genes aside from *ca1* and *ca2* were affected at high CO_2_.

### Stomatal signaling

One of the goals of this study was to identify both the short-term and longer-term responses to low CO_2_ in *ca* mutants. At the first low CO_2_ time point, expression of many genes was affected in the *ca* mutants; however, this effect was relatively small in the *ca1* mutants compared to the *ca1ca2* mutants. This result is surprising considering that *ca1* contributes to approximately 90% of bulk leaf CA activity, while *ca2* makes up only 7% of bulk leaf CA activity. The results of Studer et al. [9] and the significant differences in the temporal response of *ca1* and *ca1ca2* genotypes to low CO_2_ suggest at least a partial subfunctionalization of the two CA genes. Indeed, despite their small reduction in CA activity, *ca2* mutants have altered stomatal conductance [21]. The role of *ca2* in stomatal signaling is further supported by gene expression changes in *ca1ca2* mutants at the first low CO_2_ time point. In *Arabidopsis*, CA functions in stomatal signaling by modulating the bicarbonate concentration in guard cells in response to CO_2_. Under high CO_2_, the corresponding high bicarbonate concentration in the guard cells is sensed by a MATE transporter, RESISTANT to HIGH CO_2_ 1 (RHC1), which then binds and sequesters the kinase HIGH LEAF TEMPERATURE 1 (HT1) to the plasma membrane [27, 28]. The bound HT1 releases the kinase OPEN STOMATA1 (OST1) to trigger stomatal closure through activation of the S-type anion channel SLOW ANION CHANNEL-ASSOCIATED1 (SLAC1) [29, 30]. Since CA functions in the initial step of the CO_2_ signaling pathway through modulating bicarbonate concentrations, any effect of the *ca1* and *ca1ca2* mutants on CO_2_ signaling should be seen in differential expression of these downstream CO_2_ signaling genes. Indeed, homologs of the downstream CO_2_ signaling genes in our study were responsive to CO_2_ as expected and differentially expressed in the *ca1ca2* but not *ca1* mutants. The maize homolog of SLAC1 (GRMZM2G106921) is strongly downregulated in *ca1ca2* mutants compared to wild-type at the first low CO_2_ time point, although it returns to wild-type levels after two days at low CO_2_. The strong downregulation in *ca1ca2* mutants relative to wild-type indicates that *ca1ca2* plants adjust expression of CO_2_-sensitive stomata genes to relieve SLAC1-driven stomatal closure while adjusting for the CO_2_ signaling bottleneck due to low CA activity. The *ca1* mutants showed a similar, but non-significant, expression pattern for SLAC1. In addition to SLAC1, two isoforms of OST1 were downregulated in response to CO_2_, with more pronounced downregulation in the mutants compared to wild-type. One isoform of OST1(GRMZM2G066867) was downregulated at the first day of low CO_2_, whereas the second isoform (GRMZM2G081915) was downregulated at the second time point. Since OST1 functions in activating SLAC1 and therefore inducing stomatal closure, downregulation of OST1 would be expected to enhance stomatal opening in the mutants. Interestingly, this effect is seen at the first low CO_2_ time point. We hypothesize that the expression level is altered in response to changing CO_2_, but after acclimating to the condition over several days, the plants achieve a steady-state expression level similar to wild-type. This hypothesis would explain why altered expression levels are not observed when the plants are grown at high CO_2_, and are largely resolved after two days at low CO_2_. Although these results support a role for CA in the stomatal CO_2_ signaling pathway, there is still debate in the literature about whether the CO_2_ signal comes exclusively from the guard cells or whether it is also influenced by a mesophyll signal such as C_i_, sucrose, malate, or other metabolites [31]. It is unclear whether the changes in gene expression of stomatal genes may be affected by a mesophyll signal reflecting reduced photosynthetic rate in the mutants under low CO_2_. Additional study on these mutants may provide insight into the contribution of mesophyll and guard cell signals for stomatal responses to CO_2_.

### Photosynthetic metabolism

After two days at 9.2 Pa CO_2_, changes in photosynthetic metabolism were evident across all genotypes, but interestingly, relatively few differences were observed between the genotypes. One of our hypotheses was that CA mutant plants upregulated other isoforms of CA or enzymes in the C_4_ pathway to compensate for low CA activity. However, our RNA-seq analysis showed that none of the other CA isoforms found in leaves were upregulated in CA mutants relative to wild-type, indicating that the plants were unable to compensate for the mutation. This result is in contrast to previous work on the carbon concentrating mechanism of *Chlamydomonas reinhardtii*, which identified multiple isoforms of CA that were induced by low CO_2_ [32]. Interestingly, however, one of the predicted mitochondrial β-CAs (GRMZM2G414528) strongly increases in expression in response to CO_2_, independent of genotype. This result supports the proposed role for mitochondrial CAs in respiratory/photorespiratory CO_2_ recycling [14]. Although photorespiratory genes generally decreased at low CO_2_, many respiratory genes increased in expression at low CO_2_ (Additional file 11). Similar results have been observed for mitochondrial CA in *Chlamydomonas reinhardtii* when transitioning cells from high to low CO_2_, suggesting an anaplerotic role for mitochondrial CA [33, 34].

Photosynthetic metabolism is significantly changed by differences in the availability of atmospheric CO_2_, but surprisingly few photosynthetic genes were differentially expressed between genotypes. However, genes involved in light reactions, photorespiration, as well as the C_3_ and C_4_ cycles were all downregulated at low CO_2_, regardless of genotype. In general, the *ca* mutants tended to downregulate these genes to a larger extent than wild-type plants; although for most genes this trend was not significant after two days at low CO_2_. Interestingly, the only core C_3_ cycle gene found to be upregulated in the experiment was Rubisco activase (GRMZM2G162282) in *ca1* mutants. A similar trend was observed in *ca1ca2* mutants, although the difference was not significant (FDR = 0.087). In all genotypes, the small subunit of Rubisco was downregulated in response to low CO_2_. This indicates that under low carbon availability, plants may alter the activation state of Rubisco, while reducing the total protein content to conserve resources. Downregulation of light reaction genes is expected to account for the light stress associated with low CO_2_ availability. The downregulation of photorespiratory genes along with the C_4_ and C_3_ cycles is indicative of the precise balancing of these cycles such that photorespiration is minimized even under these low CO_2_ conditions.

### Aquaporins

Aquaporins have been shown to mediate CO_2_ diffusion in several plant species [23]. In particular, CO_2_-transporting aquaporins have been implicated in mesophyll conductance [35–37], which is defined as the movement of CO_2_ from the intercellular air spaces to the site of initial carboxylation in the mesophyll. In C_3_ plants, CA is thought to enhance mesophyll conductance directly by facilitating diffusion of CO_2_ through the chloroplast stroma [1]. Alternatively, in C_4_ plants, mesophyll conductance is defined as the movement of CO_2_ to the sites of carboxylation by PEPC, which also requires the CA-catalyzed CO_2_ hydration to bicarbonate. Previous work has demonstrated that high CA activity is important to offset low mesophyll conductance, particularly under CO_2_-limiting conditions [38, 39]. Therefore, aquaporins may play an important role in mediating CO_2_ diffusion upstream of CA and we hypothesized that CA mutants may increase expression of CO_2_-transporting aquaporins to offset low CA activity for mesophyll conductance.

Currently, the aquaporin isoforms in maize that are responsible for CO_2_ movement have not been characterized. In our study, three aquaporin genes (AC209208.3_FG002, GRMZM2G178693, GRMZM2G136032) were differentially expressed at low CO_2_ in one of the two mutant genotypes. However, these genes did not have a consistent response to CO_2_, indicating that their physiological roles likely differ. Distinct physiological roles are also supported by differences in mesophyll/bundle sheath localization. This may be reflective of some aquaporin genes being involved in CO_2_ movement, whereas others may be responding to changes in water availability due to stomatal opening at low CO_2_. Since no aquaporin was consistently upregulated in the mutant plants, our results do not support an increase in mesophyll conductance via CO_2_-transporting aquaporins as a mechanism to enhance CO_2_ availability in CA mutants.

### Carbon starvation responses

In spite of few differences between CA mutant plants and wild-type in genes enhancing CO_2_ uptake or photosynthesis, the gene expression data presents a clear pattern that the CA mutants were experiencing low carbon stress. This is consistent with previous work showing a reduction in photosynthesis under sub-ambient CO_2_ [9]. Carbon starvation responses are often characterized by changes in carbohydrate metabolism and allocation of photoassimilate into organic acids [40, 41]. Various studies have implicated trehalose-6-phosphate synthase (TPS) and associated metabolites including trehalose-6-phosphate (T6P) as a critical signal for sugar levels that potentially interacts with various other upstream and downstream pathways [42–45]. In this study, wild-type plants decreased expression of multiple isoforms of TPS at low CO_2_. This is expected, as previous studies have shown low levels of T6P in carbon-starved *Arabidopsis* seedlings [46]. In contrast, both *ca1* and *ca1ca2* mutants either maintain relatively constant TPS expression across all CO_2_ conditions, or even upregulate TPS at low CO_2_. This pattern is somewhat paradoxical given other evidence suggesting carbon starvation in CA mutant plants; however, we hypothesize that CA mutant plants utilize TPS to reprogram carbohydrate and organic acid metabolism in response to the extreme carbon starvation they experience at low CO_2_.

Many studies have linked TPS to reprogramming of carbohydrate metabolism, particularly in the context of carbon starvation. Early work on T6P and sugar signaling in *Arabidopsis* led to the conclusion that T6P is critical for proper use of carbohydrates and growth [43]. Schluepmann et al. [43] discussed their findings in the context of similar work in yeast, and hypothesized that T6P may have evolved as a way to regulate glycolysis to handle sudden and large changes in carbohydrate availability, contributing to organisms’ versatility in handling diverse environmental conditions. Since then, a clear link between T6P, sucrose, and organic acid metabolism has emerged [45]. Specifically, T6P appears to act both as a sugar signal as well as a negative feedback regulator of sucrose; high levels of sucrose lead to an increase of T6P, which in turn stimulates consumption of sucrose [47]. Furthermore, recent work has also shown that induction of TPS is associated with altered partitioning of photoassimilate, promoting carbon flux into organic acids [45]. The mechanism linking TPS to organic acids is less well understood, although there is some evidence that T6P may activate nitrate reductase, affecting nitrogen uptake, or PEPC, affecting respiratory flux [45]. Yet additional studies have suggested a role for T6P in stomatal opening [44]. Our results suggest that *ca1* and *ca1ca2* mutants do not regulate TPS as expected for carbon starvation. Instead, the high levels of TPS, expected to confer high levels of T6P, may indicate a reprogramming of primary metabolism in mutant plants, stimulating sucrose consumption for growth and altering photoassimilate partitioning into organic acids with high N:C to conserve carbon resources.

Investment in high N:C organic acids are a classic symptom of carbon starvation, as observed in several metabolite studies [40, 41]. These organic acids typically include asparagine and arginine, although results have been inconsistent between studies. In our study, two genes encoding asparagine synthetase were strongly upregulated, as well as aspartate aminotransferase which is an essential step in synthesizing asparagine. In addition, asparaginase (an enzyme which degrades asparagine) was downregulated and homologs of transcription factors characterized in *Arabidopsis* as regulating asparagine synthetase were upregulated. Furthermore, the isoform of aspartate aminotransferase upregulated in this experiment is not expected to have a role in C_4_ photosynthesis; in contrast, the C_4_ isoform was strongly downregulated at low CO_2_. The upregulation of multiple genes with putative functions in amino acid transport also suggests that plants were actively engaging in remobilization of nitrogen in response to low CO_2_. Overall, these findings are consistent with an investment in amino acids with a high N:C ratio for remobilization of nitrogen. Although recent work in *Arabidopsis* has suggested a role for CA in amino acid metabolism [19], we did not observe significant differences in the biosynthesis of other amino acids. Therefore, our results are most consistent with a remobilization of nitrogen in energy efficient amino acids, rather than a direct role for CA in amino acid synthesis.

## Conclusions

RNA-seq analysis of *ca1* and *ca1ca2* mutant plants in response to low CO_2_ found that despite experiencing reductions in photosynthetic capacity under low CO_2_ due to CA limitation, CA mutant plants do not compensate by upregulating other CA genes or genes involved in CO_2_ uptake or assimilation. However, CA mutant plants had altered expression of genes in the CO_2_ stomatal signaling pathway consistent with previous work demonstrating abnormal stomatal responses in CA mutants. These findings support a similar CO_2_ signaling pathway in maize as was previously described in the C_3_ dicot *Arabidopsis thaliana.* CA mutant plants also exhibited typical signs of carbon starvation, including sugar signaling and amino acid metabolism. This work identified previously uncharacterized transcription factors which may function to regulate metabolic responses to carbon starvation. Future work on the other isoforms of CA will provide additional insight into the role of CA in photosynthesis and stomatal signaling in maize.

## Methods

### Plant growth and sampling

Maize lines carrying the mutant alleles *ca1-m1::Ds* and *ca1-d1ca2-m1::Ds* (generated and provided by A. Studer; previously described in [9]) in a W22 background were crossed to the reference line B73. These F1 plants were then backcrossed to B73 for two additional generations prior to selfing. This resulted in BC2S1 kernels segregating wild-type and *ca1* and *ca1ca2* mutant alleles. Kernels were germinated and grown in deep-well plug trays (Hummert #11–8606-1), in a controlled-environment growth chamber (Biochambers; TPC-19) at a photosynthetic photon flux density of 500 μmol m^− 2^ s^− 1^ at plant height, approximately 50% relative humidity, air temperature of 31 °C/22 °C day/night with a 16-h day. On the 7th day after planting (DAP), homozygous and wild-type seedlings were identified using the PCR assay described in Studer et al. [9]. The *p*CO_2_ in the chamber was initially 920 Pa (10,000 ppm) during the photoperiod. On the 12th DAP, the *p*CO_2_ in the chamber was reduced to 9.2 Pa (100 ppm) in the final hours of the photoperiod and maintained during the photoperiod for the remainder of the experiment.

Leaf samples for RNA were taken three hours after the beginning of the photoperiod on days 12, 13 and 15. Each genotype/time-point combination was sampled in triplicate, with each of these three biological replicates containing a pool of leaves from four individual plants. Leaf samples were immediately frozen in liquid nitrogen and stored at − 80 °C until extraction.

### RNA extraction and library preparation

Leaf tissue was ground in a Fluid Management Harbil 5G-HD paint shaker. Total RNA from the pooled samples of each line was isolated using TriPure reagent according to manufacturer’s instructions (Invitrogen), and resuspended in 1x RNA Secure solution (Ambion). Library preparation was performed according to the strand-specific protocol developed by Wang et al. [48], resulting in three libraries per genotype/time-point combination for a total of 36 libraries. Libraries were sequenced on two lanes of an Illumina HiSeq2000 (Cornell University), generating 50-bp single-end reads. All libraries were sequenced on both lanes to account for lane effects.

### RNA-seq analysis

Raw Illumina reads from RNA-seq libraries were de-multiplexed using standard Casava settings. Reads were trimmed using TrimGalore [49] with a stringency of 2 and default settings. Trimmed reads were aligned to the maize reference genome B73 AGPv3 (Ensembl) using Bowtie v2.2.5.0 [50] and TopHat2 v2.1.0 [51]. Minimum and maximum intron length were 5 and 60,000 bp, respectively; all other parameters were set to default values. Raw read counts were generated using htseq-count in union mode [52]. After verifying no significant effect of sequencing lane by principal component analysis, the data from each lane were merged in subsequent steps. Lowly expressed genes were filtered using edgeR [53], keeping genes with counts-per-million > 1 in at least three samples. Normalization factors for library sizes were calculated using standard methods. The quasi-likelihood negative binomial generalized log-linear model in edgeR (glmQLFit) was used to fit a model with two sets of mutants and two wild-types as treatments, while downstream a priori contrasts were conducted to statistically test differences in expression between groups of interest. Gene ontology enrichment was evaluated using the R package GOstats [54]. RNA-seq data have been deposited in the NCBI SRA (SRP133928).

Sequencing of biological triplicates for all genotypes at each time point yielded on average 30.8 ± 1.0 million reads per genotype:treatment combination. After quality control and adapter trimming, an average of 95.5 ± 0.1% reads mapped to the B73 reference genome. Differential expression was determined at a false-discovery rate of 0.05.

### qRT-PCR

Quantitative real-time PCR was performed on 12 genes which were either differentially expressed or showed a CO_2_ response in the RNA-seq data. These included SLAC1, three aquaporins, aspartate aminotransferase, two asparagine synthetases, PEPC, TPS, and three transcription factors. Gene IDs and primer sequences are provided in Additional file 12. Primers for each gene were designed using Roche’s Universal ProbeLibrary Assay Design center. The original RNA samples were treated using DNaseI (NEB M0303) according to manufacturer’s instructions followed by an ethanol precipitation, and quantified with a Qubit fluorometer (Invitrogen). cDNA synthesis was performed using the qScript cDNA synthesis kit (Quantabio) according to manufacturer’s instructions with 1 μg of DNase-treated RNA. The reference gene for normalization (GRMZM2G046402) was selected using the leaf gradient RNA-seq data from Wang et al. [55] because it did not vary with leaf development. Expression of this gene from RNA-seq was consistent across all samples, supporting its use as a reference gene.

qPCR was performed on a QuantStudio 7 Real-Time PCR System (Applied Biosystems) using qScript cDNA SuperMix (Quantabio) and standard cycling conditions. Standard curves were generated from a 7-point serial dilution for each primer set to determine assay efficiency and analyzed using the Applied Biosystems Standard Curve Analysis Module. Estimated efficiency was between 90 and 110% for all primer sets. Expression levels were estimated using the relative standard curve method. Reactions were performed in triplicate, and technical replicates were averaged prior to normalizing by the reference gene. For one gene, GRMZM2G078472, several samples did not amplify during the 40-cycle qPCR run. This is expected given the low expression levels observed in the RNA-seq data. In these cases, a Cq value of 40 was imputed to allow for calculations of log_2_-fold change.

## Additional files

Additional file 1: Gene ontology terms of differentially expressed genes in ca1 and ca1ca2 mutants after two days at low CO2. GO term enrichment was calculated using the R package GOStats with a *p*-value cutoff of 0.05. Each sheet corresponds to ontology terms for Biological Process (BP), Cellular Compartment (CC), Molecular Function (MF) or KEGG. (XLSX 33 kb)

Additional file 2: Screenshot of IGV showing reads aligning to *Ca3* (annotated as the second half of GRMZM2G348512) at Low2. No up-regulation of *Ca3* is observed. The appearance of down-regulation in the CA mutants is likely reflective of the high sequence homology between *Ca1, Ca2,* and *Ca3*. In the mutants, fewer *Ca1* and *Ca2* transcripts are present, leading to fewer misaligned reads to *Ca3. (PDF 83 kb)*

Additional file 3: Photosynthetic gene expression relative to wild-type in each genotype at each CO_2_. The heatmap is broken into three groups: transporters, C_4_ genes, and C_3_ genes. Colors indicate log-fold change relative to wild-type. Significant differences are indicated with an asterisk (*). The relative expression level is given on the right of the heatmap, indicating the log_2_(counts-per-million) of wild-type plants at Low2. (PDF 127 kb)

Additional file 4: Differentially expressed genes involved in cell wall and carbohydrate metabolism, and genes encoding aquaporins. The average counts-per-million (CPM), normalized by edgeR, for wild-type plants is given for each gene and CO2 condition. The log-fold change (logFC) and false-discovery rate (FDR) are given for differential expression between mutant and wild-type at each CO2 condition. (XLSX 19 kb)

Additional file 5: Trehalose-6-phosphate synthase (TPS) expression relative to wild-type in each genotype at each CO_2_. Gene names are as described in Henry et al. (2014). Colors indicate log-fold change relative to wild-type. Significant differences are indicated with an asterisk (*). The relative expression level is given on the right of the heatmap, indicating the log_2_(counts-per-million) of wild-type plants at Low2. (PDF 96 kb)

Additional file 6: Gene IDs for Fig. 3. The average counts-per-million (CPM), normalized by edgeR, for wild-type plants is given for each gene and CO_2_ condition. The log-fold change (logFC) and false-discovery rate (FDR) are given for differential expression between mutant and wild-type at each CO_2_ condition. (XLSX 13 kb)

Additional file 7: Transcription factors shown in Fig. 4 with family classification. The log-fold change (logFC) and false-discovery rate (FDR) are given for the differential expression analysis of *ca1ca2* mutant vs. wild-type. (XLSX 17 kb)

Additional file 8: The log_2_-fold change of mutants relative to wild-type calculated with qRT-PCR and RNA-seq. (PDF 548 kb)

Additional file 9: Correlation between log_2_-fold change values generated with qRT-PCR and RNA-seq. Points indicate averaged log_2_-fold change across biological replicates for a given genotype/condition. (PDF 157 kb)

Additional file 10: Relative expression measured by qRT-PCR showing the CO_2_ response of 12 measured genes. Values are presented as means ± standard error. (PDF 985 kb)

Additional file 11: Differential expression analysis (mutant vs. wild-type) providing the log-fold change and false-discovery rate for all expressed genes in each CO_2_ condition. (XLSX 3040 kb)

Additional file 12: Primer sequences and IDs of genes selected for qRT-PCR validation. (XLSX 9 kb)

## Abbreviations

CA: Carbonic anhydrase
GO: gene ontology
HT1: HIGH LEAF TEMPERATURE 1
OST1: OPEN STOMATA 1
PEPC: Phospho*enol*pyruvate Carboxylase
RHC1: RESISTANT to HIGH CO_2_ 1
Rubisco: Ribulose-1,5-bisphosphate carboxylase/oxygenase
SLAC1: SLOW ANION CHANNEL-ASSOCIATED 1
T6P: Trehalose-6-phosphate
TPS: Trehalose-6-phosphate synthase

## References

[1] Badger MR, Price GD (1994). The role of carbonic anhydrase in photosynthesis. Annu Rev Plant Physiol Plant Mol Biol.

[2] Ludwig M (2012). Carbonic anhydrase and the molecular evolution of C_4_ photosynthesis. Plant Cell Environ.

[3] Price GD, von Caemmerer S, Evans JR, Yu J, Lloyd J, Oja V, Kell P, Harrison K, Gallagher A, Badger MR (1994). Specific reduction of chloroplast carbonic anhydrase activity by antisense RNA in transgenic tobacco plants has a minor effect on photosynthetic CO_2_ assimilation. Planta.

[4] Williams TG, Flanagan LB, Coleman JR (1996). Photosynthetic gas exchange and discrimination against ^13^CO_2_ and C^18^O^16^O in tobacco plants modified by an antisense construct to have low chloroplastic carbonic anhydrase. Plant Physiol.

[5] Hatch M, Burnell J (1990). Carbonic anhydrase activity in leaves and its role in the first step of C_4_ photosynthesis. Plant Physiol.

[6] von Caemmerer S, Quinn V, Hancock NC, Price GD, Furbank RT, Ludwig M (2004). Carbonic anhydrase and C_4_ photosynthesis: a transgenic analysis. Plant Cell Environ.

[7] Cousins AB, Badger MR, von Caemmerer S (2006). Carbonic anhydrase and its influence on carbon isotope discrimination during C_4_ photosynthesis. Insights from antisense RNA in *Flaveria bidentis*. Plant Physiol.

[8] Gillon JS, Yakir D (2000). Naturally low carbonic anhydrase activity in C_4_ and C_3_ plants limits discrimination against C^18^OO during photosynthesis. Plant Cell Environ.

[9] Studer AJ, Gandin A, Kolbe AR, Wang L, Cousins AB, Brutnell TP (2014). A limited role for carbonic anhydrase in C_4_ photosynthesis as revealed by a *ca1ca2* double mutant in maize. Plant Physiol.

[10] Osborn HL, Alonso-Cantabrana H, Sharwood RE, Covshoff S, Evans JR, Furbank RT, von Caemmerer S (2017). Effects of reduced carbonic anhydrase activity on CO_2_ assimilation rates in *Setaria viridis*: a transgenic analysis. J Exp Bot.

[11] DiMario RJ, Clayton H, Mukherjee A, Ludwig M, Moroney JV (2017). Plant carbonic anhydrases - structures, locations, evolution and physiological roles. Mol Plant.

[12] Perales M, Eubel H, Heinemeyer J, Colaneri A, Zabaleta E, Braun HP (2005). Disruption of a nuclear gene encoding a mitochondrial gamma carbonic anhydrase reduces complex I and supercomplex I + III_2_ levels and alters mitochondrial physiology in *Arabidopsis*. J Mol Biol.

[13] Fromm S, Senkler J, Zabaleta E, Peterhansel C, Braun HP (2016). The carbonic anhydrase domain of plant mitochondrial complex I. Physiol Plant.

[14] Zabaleta E, Martin MV, Braun HP (2012). A basal carbon concentrating mechanism in plants?. Plant Sci.

[15] Hoang CV, Chapman KD (2002). Biochemical and molecular inhibition of plastidial carbonic anhydrase reduces the incorporation of acetate into lipids in cotton embryos and tobacco cell suspensions and leaves. Plant Physiol.

[16] Hu H, Boisson-Dernier A, Israelsson-Nordstrom M, Bohmer M, Xue S, Ries A, Godoski J, Kuhn JM, Schroeder JI (2010). Carbonic anhydrases are upstream regulators of CO_2_-controlled stomatal movements in guard cells. Nat Cell Biol.

[17] Chen T, Wu H, Wu J, Fan X, Li X, Lin Y (2017). Absence of OsβCA1 causes a CO_2_ deficit and affects leaf photosynthesis and the stomatal response to CO_2_ in rice. Plant J.

[18] Martin V, Villarreal F, Miras I, Navaza A, Haouz A, Gonzalez-Lebrero RM, Kaufman SB, Zabaleta E (2009). Recombinant plant gamma carbonic anhydrase homotrimers bind inorganic carbon. FEBS Lett.

[19] DiMario RJ, Quebedeaux JC, Longstreth DJ, Dassanayake M, Hartman MM, Moroney JV (2016). The cytoplasmic carbonic anhydrases βCA2 and βCA4 are required for optimal plant growth at low CO_2_. Plant Physiol.

[20] Zhang N, Gibon Y, Wallace JG, Lepak N, Li P, Dedow L, Chen C, So YS, Kremling K, Bradbury PJ (2015). Genome-wide association of carbon and nitrogen metabolism in the maize nested association mapping population. Plant Physiol.

[21] Kolbe AR, Brutnell TP, Cousins AB, Studer AJ (2018). Carbonic anhydrase mutants in *Zea mays* have altered stomatal responses to environmental signals. Plant Physiol.

[22] Kolbe AR, Studer AJ, Cousins AB (2018). Biochemical and transcriptomic analysis of maize diversity to elucidate drivers of leaf carbon isotope composition. Funct Plant Biol.

[23] Heinen RB, Ye Q, Chaumont F (2009). Role of aquaporins in leaf physiology. J Exp Bot.

[24] Li P, Ponnala L, Gandotra N, Wang L, Si Y, Tausta SL, Kebrom TH, Provart N, Patel R, Myers CR (2010). The developmental dynamics of the maize leaf transcriptome. Nat Genet.

[25] Subbaiah CC, Palaniappan A, Duncan K, Rhoads DM, Huber SC, Sachs MM (2006). Mitochondrial localization and putative signaling function of sucrose synthase in maize. J Biol Chem.

[26] Yilmaz A, Nishiyama MY, Fuentes BG, Souza GM, Janies D, Gray J, Grotewold E (2009). GRASSIUS: a platform for comparative regulatory genomics across the grasses. Plant Physiol.

[27] Hashimoto M, Negi J, Young J, Israelsson M, Schroeder JI, Iba K (2006). Arabidopsis HT1 kinase controls stomatal movements in response to CO_2_. Nat Cell Biol.

[28] Tian W, Hou C, Ren Z, Pan Y, Jia J, Zhang H, Bai F, Zhang P, Zhu H, He Y (2015). A molecular pathway for CO(2) response in Arabidopsis guard cells. Nat Commun.

[29] Negi J, Matsuda O, Nagasawa T, Oba Y, Takahashi H, Kawai-Yamada M, Uchimiya H, Hashimoto M, Iba K (2008). CO_2_ regulator SLAC1 and its homologues are essential for anion homeostasis in plant cells. Nature.

[30] Vahisalu T, Kollist H, Wang YF, Nishimura N, Chan WY, Valerio G, Lamminmaki A, Brosche M, Moldau H, Desikan R (2008). SLAC1 is required for plant guard cell S-type anion channel function in stomatal signalling. Nature.

[31] Lawson T, Simkin AJ, Kelly G, Granot D (2014). Mesophyll photosynthesis and guard cell metabolism impacts on stomatal behaviour. New Phytol.

[32] Moroney JV, Ma Y, Frey WD, Fusilier KA, Pham TT, Simms TA, DiMario RJ, Yang J, Mukherjee B (2011). The carbonic anhydrase isoforms of *Chlamydomonas reinhardtii:* intracellular location, expression, and physiological roles. Photosynthesis Res.

[33] Giordano M, Norici A, Forssen M, Eriksson M, Raven JA (2003). An anaplerotic role for mitochondrial carbonic anhydrase in *Chlamydomonas reinhardtii*. Plant Physiol.

[34] Eriksson M, Karlsson J, Ramazanov Z, Gardestrom P, Samuelsson G (1996). Discovery of an algal mitochondrial carbonic anhydrase: molecular cloning and characterization of a low-CO_2_ induced polypeptide in *Chlamydomonas reinhardtii*. Proc Natl Acad Sci U S A.

[35] Hanba YT, Shibasaka M, Hayashi Y, Hayakawa T, Kasamo K, Terashima I, Katsuhara M (2004). Overexpression of the barley aquaporin HvPIP2;1 increases internal CO_2_ conductance and CO_2_ assimilation in the leaves of transgenic rice. Plant Cell Physiol.

[36] Flexas J, Ribas-Carbo M, Hanson DT, Bota J, Otto B, Cifre J, McDowell N, Medrano H, Kaldenhoff R. Tobacco aquaporin NtAQP1 is involved in mesophyll conductance to CO_2_ in vivo. Plant J. 2006;48(3):427–39. 10.1111/j.1365-313X.2006.02879.x 17010114

[37] Uehlein N, Sperling H, Heckwolf M, Kaldenhoff R (2012). The *Arabidopsis* aquaporin *PIP1;2* rules cellular CO_2_ uptake. Plant Cell Environ.

[38] Ubierna N, Gandin A, Boyd RA, Cousins AB (2017). Temperature response of mesophyll conductance in three C_4_ species calculated with two methods: ^18^O discrimination and *in vitro* V_pmax_. New Phytol.

[39] Kolbe AR, Cousins AB (2018). Mesophyll conductance in Zea mays responds transiently to CO_2_ availability: implications for transpiration efficiency in C_4_ crops. New Phytol.

[40] Usadel B, Blasing OE, Gibon Y, Retzlaff K, Hohne M, Gunther M, Stitt M (2008). Global transcript levels respond to small changes of the carbon status during progressive exhaustion of carbohydrates in Arabidopsis rosettes. Plant Physiol.

[41] Czedik-Eysenberg A, Arrivault S, Lohse MA, Feil R, Krohn N, Encke B, Nunes-Nesi A, Fernie AR, Lunn JE, Sulpice R (2016). The interplay between carbon availability and growth in different zones of the growing maize leaf. Plant Physiol.

[42] Kolbe A, Tiessen A, Schluepmann H, Paul M, Ulrich S, Geigenberger P (2005). Trehalose 6-phosphate regulates starch synthesis via posttranslational redox activation of ADP-glucose pyrophosphorylase. Proc Natl Acad Sci U S A.

[43] Schluepmann H, Pellny TK, van Dijken A, Smeekens S, Paul M (2003). Trehalose 6-phosphate is indispensable for carbohydrate utilization and growth in *Arabidopsis thaliana*. Proc Natl Acad Sci U S A.

[44] Figueroa CM, Lunn JE (2016). A tale of two sugars: Trehalose 6-phosphate and sucrose. Plant Physiol.

[45] Figueroa CM, Feil R, Ishihara H, Watanabe M, Kolling K, Krause U, Hohne M, Encke B, Plaxton WC, Zeeman SC (2016). Trehalose 6-phosphate coordinates organic and amino acid metabolism with carbon availability. Plant J.

[46] Lunn JE, Feil R, Hendriks JH, Gibon Y, Morcuende R, Osuna D, Scheible WR, Carillo P, Hajirezaei MR, Stitt M (2006). Sugar-induced increases in trehalose 6-phosphate are correlated with redox activation of ADPglucose pyrophosphorylase and higher rates of starch synthesis in Arabidopsis thaliana. Biochem J.

[47] Yadav UP, Ivakov A, Feil R, Duan GY, Walther D, Giavalisco P, Piques M, Carillo P, Hubberten HM, Stitt M (2014). The sucrose-trehalose 6-phosphate (Tre6P) nexus: specificity and mechanisms of sucrose signalling by Tre6P. J Exp Bot.

[48] Wang L, Si Y, Dedow LK, Shao Y, Liu P, Brutnell TP (2011). A low-cost library construction protocol and data analysis pipeline for Illumina-based strand-specific multiplex RNA-seq. PLoS One.

[49] Trim Galore! [https://www.bioinformatics.babraham.ac.uk/projects/trim_galore/]. Accessed 9 Oct 2014.

[50] Langmead B, Salzberg SL (2012). Fast gapped-read alignment with bowtie 2. Nat Methods.

[51] Kim D, Pertea G, Trapnell C, Pimentel H, Kelley R, Salzberg SL (2013). TopHat2: accurate alignment of transcriptomes in the presence of insertions, deletions, and gene fusions. Genome Biol.

[52] Anders S, Pyl PT, Huber W (2015). HTSeq--a Python framework to work with high-throughput sequencing data. Bioinformatics.

[53] Robinson MD, McCarthy DJ, Smyth GK (2010). edgeR: a Bioconductor package for differential expression analysis of digital gene expression data. Bioinformatics.

[54] Falcon S, Gentleman R (2007). Using GOstats to test gene lists for GO term association. Bioinformatics.

[55] Wang L, Czedik-Eysenberg A, Mertz RA, Si Y, Tohge T, Nunes-Nesi A, Arrivault S, Dedow LK, Bryant DW, Zhou W (2014). Comparative analyses of C_4_ and C_3_ photosynthesis in developing leaves of maize and rice. Nat Biotechnol.

