# Supplementary material for: Insights from transcriptome profiling on the non-photosynthetic and stomatal signaling response of maize carbonic anhydrase mutants to low CO2
Source: BMC Genomics. 2019 Feb 15;20:138. doi: 10.1186/s12864-019-5522-7 (PMC6377783; doi:10.1186/s12864-019-5522-7)
Supplement: Supplementary file 2 — Screenshot of IGV showing reads aligning to Ca3 (annotated as the second half of GRMZM2G348512) at Low2. No up-regulation of Ca3 is observed. The appearance of down-regulation in the CA mutants is likely reflective of the high sequence homology between Ca1, Ca2, and Ca3. In the mutants, fewer Ca1 and Ca2 transcripts are present, leading to fewer misaligned reads to Ca3. (PDF 83 kb) [file 12864_2019_5522_MOESM2_ESM.pdf]

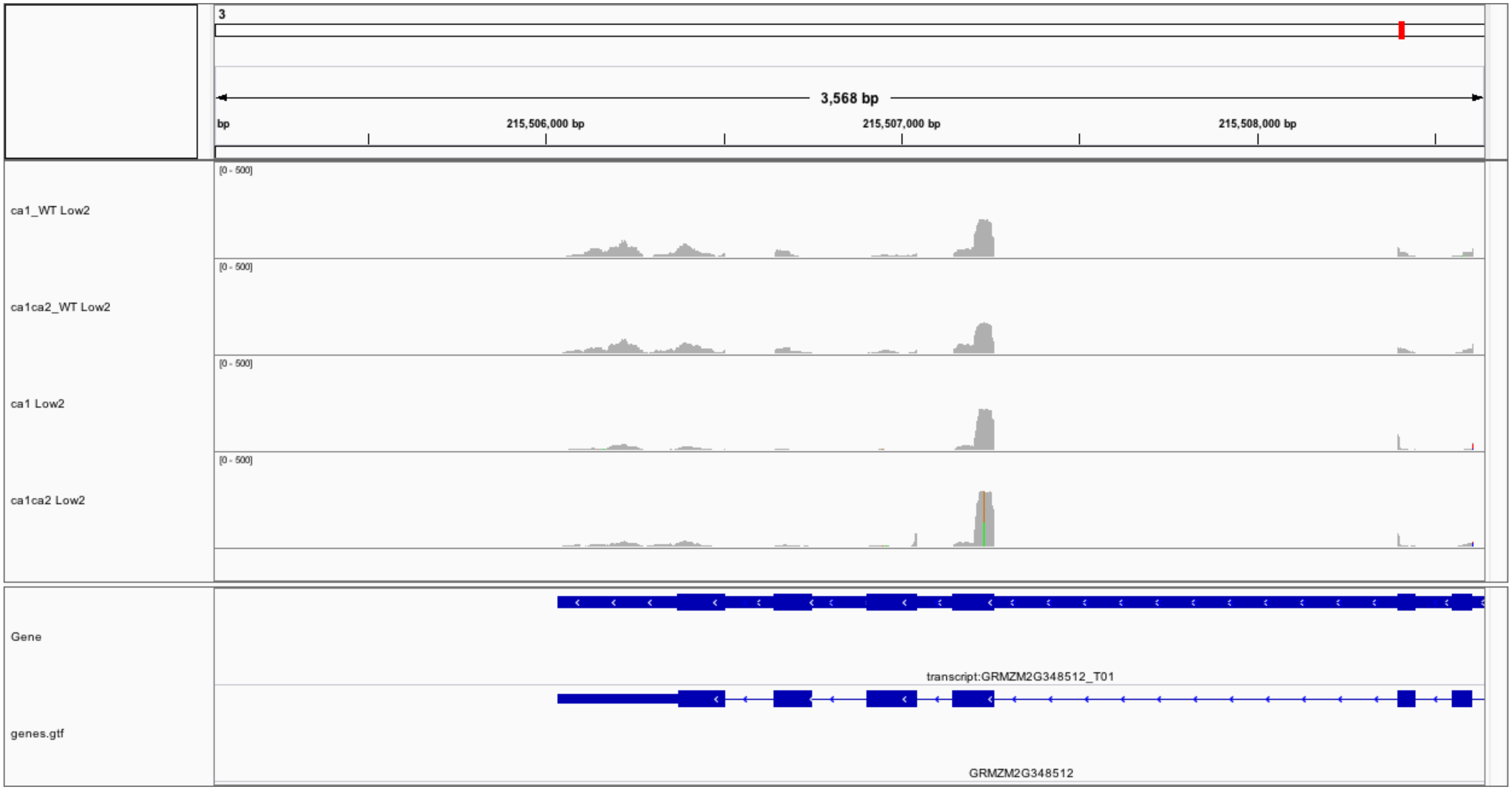

Additional file 2: Screenshot of IGV showing reads aligning to *Ca3* (annotated as the second half of GRMZM2G348512) at Low2. No up-regulation of *Ca3* is observed. The appearance of down-regulation in the CA mutants is likely reflective of the high sequence homology between *Ca1, Ca2,* and *Ca3*. In the mutants, fewer *Ca1* and *Ca2* transcripts are present, leading to fewer misaligned reads to *Ca3*.
