# Supplementary material for: Insights from transcriptome profiling on the non-photosynthetic and stomatal signaling response of maize carbonic anhydrase mutants to low CO2
Source: BMC Genomics. 2019 Feb 15;20:138. doi: 10.1186/s12864-019-5522-7 (PMC6377783; doi:10.1186/s12864-019-5522-7)
Supplement: Supplementary file 3 — Photosynthetic gene expression relative to wild-type in each genotype at each CO2. The heatmap is broken into three groups: transporters, C4 genes, and C3 genes. Colors indicate log-fold change relative to wild-type. Significant differences are indicated with an asterisk (*). The relative expression level is given on the right of the heatmap, indicating the log2(counts-per-million) of wild-type plants at Low2. (PDF 127 kb) [file 12864_2019_5522_MOESM3_ESM.pdf]

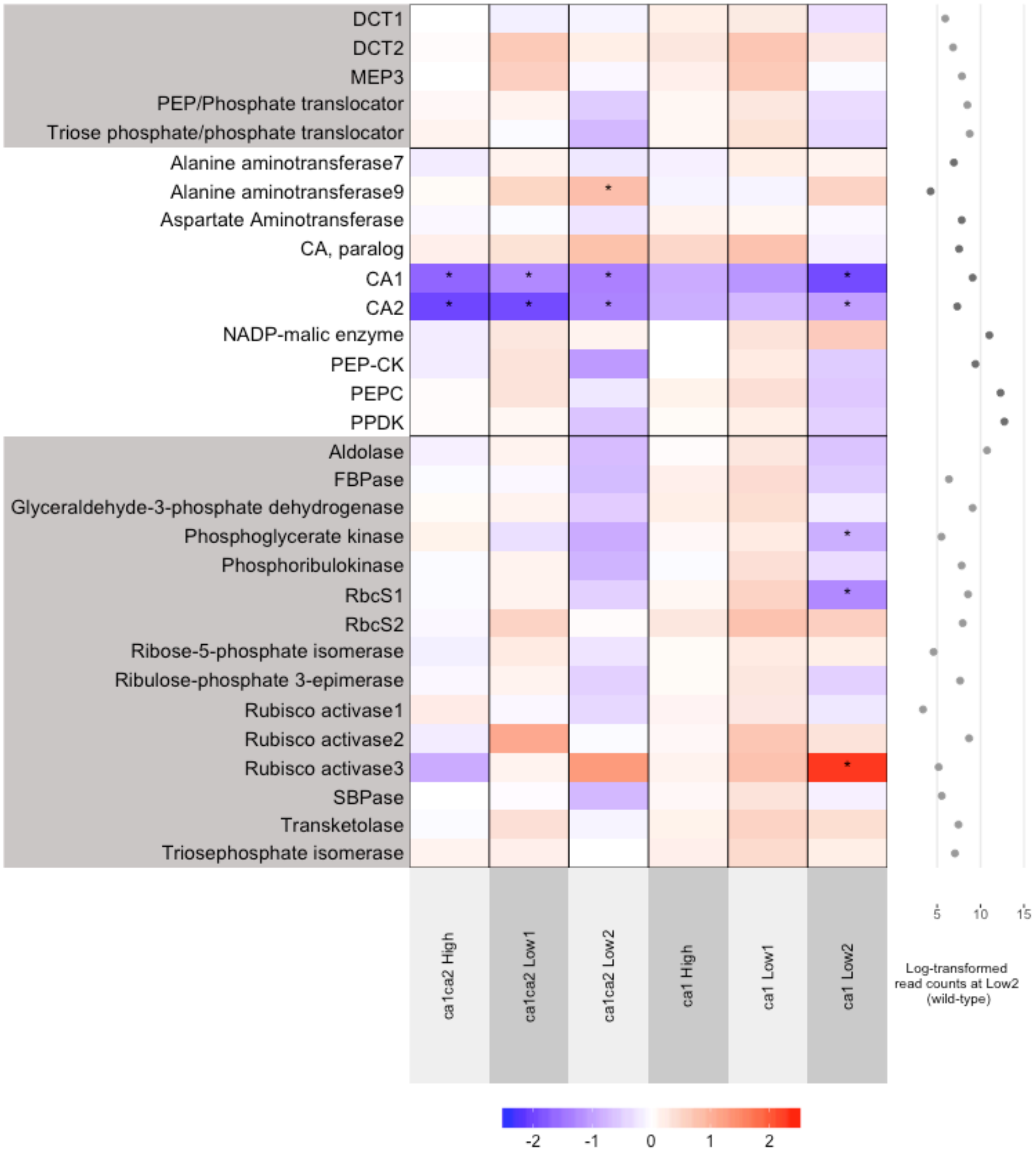

Additional file 3: Photosynthetic gene expression relative to wild-type in each genotype at each CO_2. The heatmap is broken into three groups: transporters, C_4 genes, and C_3 genes. Colors indicate log-fold change relative to wild-type. Significant differences are indicated with an asterisk (*). The relative expression level is given on the right of the heatmap, indicating the log_2(counts-per-million) of wild-type plants at Low2.
