# Supplementary material for: Insights from transcriptome profiling on the non-photosynthetic and stomatal signaling response of maize carbonic anhydrase mutants to low CO2
Source: BMC Genomics. 2019 Feb 15;20:138. doi: 10.1186/s12864-019-5522-7 (PMC6377783; doi:10.1186/s12864-019-5522-7)
Supplement: Supplementary file 8 — The log2-fold change of mutants relative to wild-type calculated with qRT-PCR and RNA-seq. (PDF 548 kb) [file 12864_2019_5522_MOESM8_ESM.pdf]

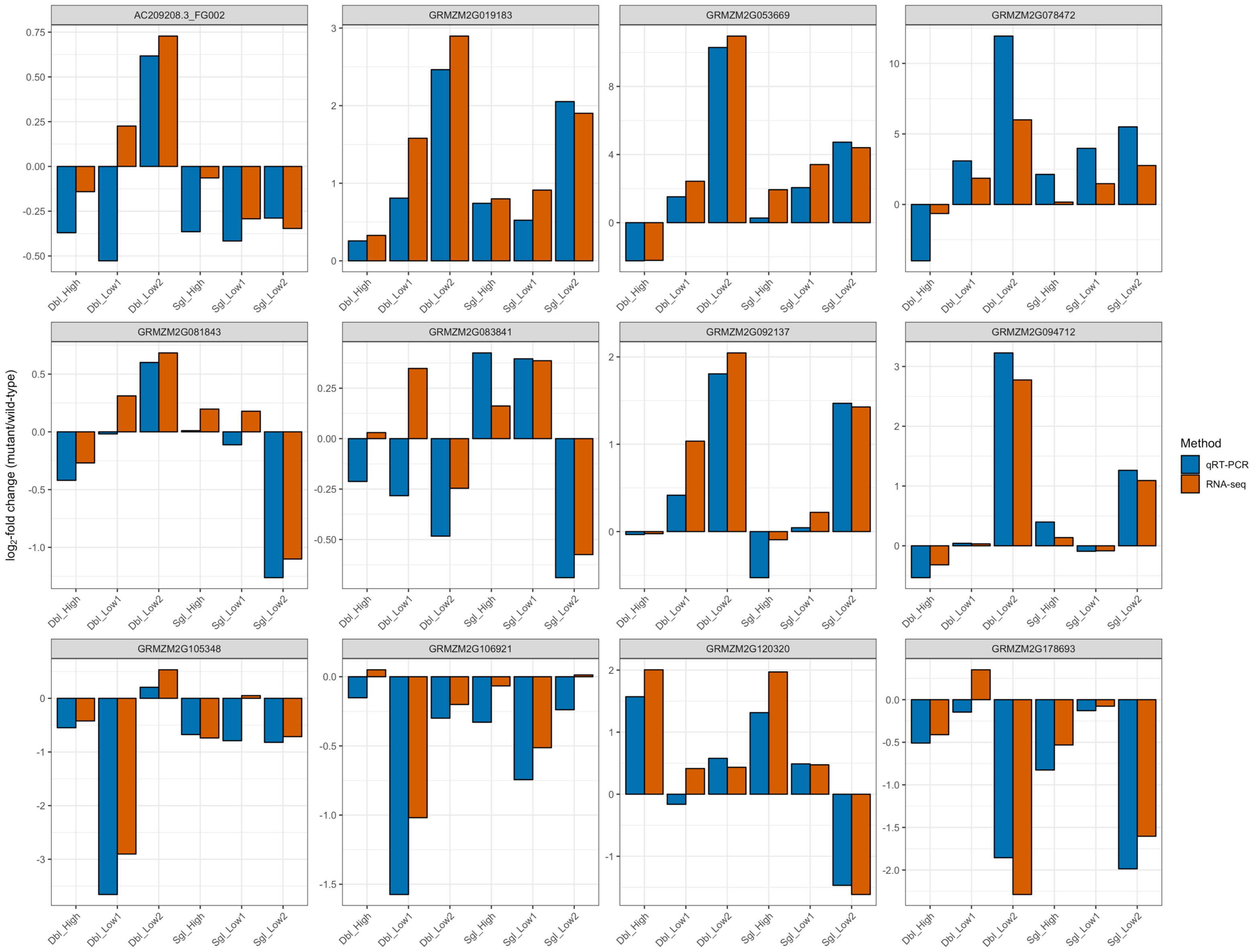

Additional file 8: The $\log_2$-fold change of mutants relative to wild-type calculated with qRT-PCR and RNA-seq.
