# Supplementary material for: Insights from transcriptome profiling on the non-photosynthetic and stomatal signaling response of maize carbonic anhydrase mutants to low CO2
Source: BMC Genomics. 2019 Feb 15;20:138. doi: 10.1186/s12864-019-5522-7 (PMC6377783; doi:10.1186/s12864-019-5522-7)
Supplement: Supplementary file 9 — Correlation between log2-fold change values generated with qRT-PCR and RNA-seq. Points indicate averaged log2-fold change across biological replicates for a given genotype/condition. (PDF 157 kb) [file 12864_2019_5522_MOESM9_ESM.pdf]

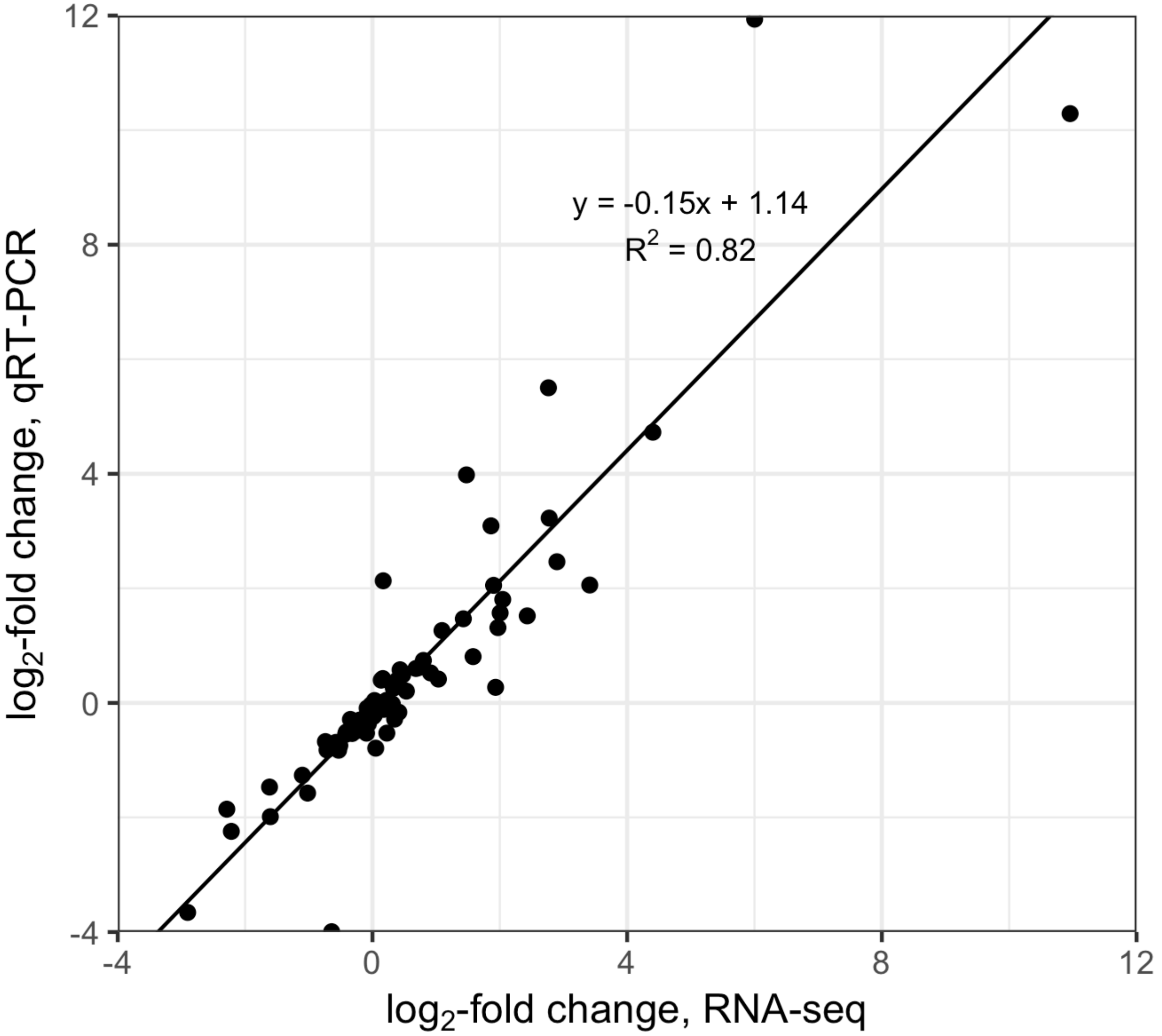

Additional file 9: Correlation between $\log_2$-fold change values generated with qRT-PCR and RNA-seq. Points indicate averaged $\log_2$-fold change across biological replicates for a given genotype/condition.
