# Supplementary material for: Insights from transcriptome profiling on the non-photosynthetic and stomatal signaling response of maize carbonic anhydrase mutants to low CO2
Source: BMC Genomics. 2019 Feb 15;20:138. doi: 10.1186/s12864-019-5522-7 (PMC6377783; doi:10.1186/s12864-019-5522-7)
Supplement: Supplementary file 10 — Relative expression measured by qRT-PCR showing the CO2 response of 12 measured genes. Values are presented as means ± standard error. (PDF 985 kb) [file 12864_2019_5522_MOESM10_ESM.pdf]

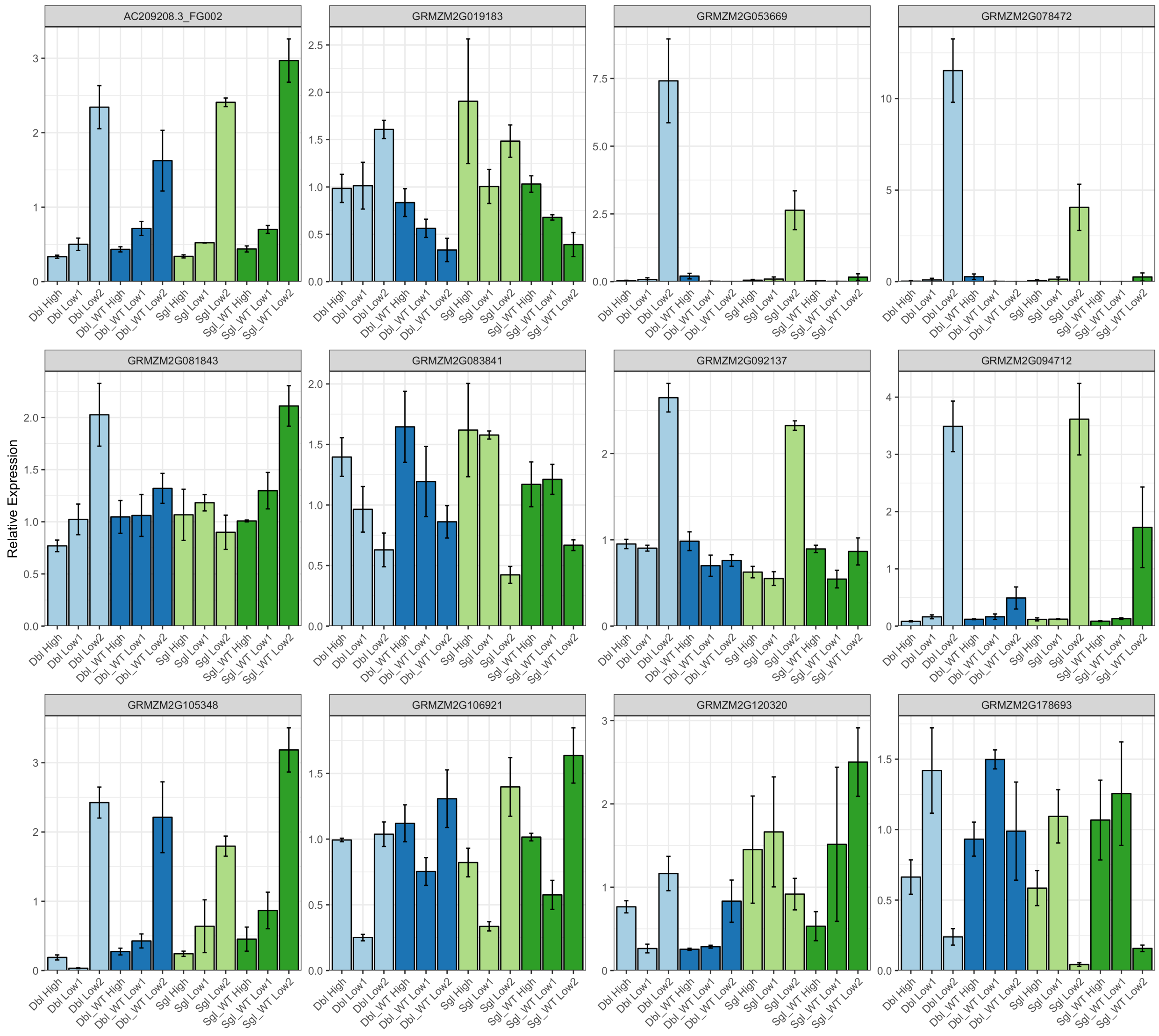

Additional file 10: Relative expression measured by qRT-PCR showing the CO_2 response of 12 measured genes. Values are presented as means ± standard error.
